# Spatial and temporal distribution of visual information coding in lateral prefrontal cortex

**DOI:** 10.1111/ejn.12754

**Published:** 2014-10-11

**Authors:** Mikiko Kadohisa, Makoto Kusunoki, Philippe Petrov, Natasha Sigala, Mark J Buckley, David Gaffan, John Duncan

**Affiliations:** 1 MRC Cognition and Brain Sciences UnitCambridge, UK; 2 Department of Experimental Psychology, University of OxfordSouth Parks Rd, Oxford, OX1 3UD, UK; 3 Brighton and Sussex Medical School, University of SussexBrighton, UK; 4 Sackler Centre for Consciousness Science, University of SussexBrighton, UK

**Keywords:** behaving monkey, dynamic coding, frontal specialisation, prefrontal cortex

## Abstract

Prefrontal neurons code many kinds of behaviourally relevant visual information. In behaving monkeys, we used a cued target detection task to address coding of objects, behavioural categories and spatial locations, examining the temporal evolution of neural activity across dorsal and ventral regions of the lateral prefrontal cortex (encompassing parts of areas 9, 46, 45A and 8A), and across the two cerebral hemispheres. Within each hemisphere there was little evidence for regional specialisation, with neurons in dorsal and ventral regions showing closely similar patterns of selectivity for objects, categories and locations. For a stimulus in either visual field, however, there was a strong and temporally specific difference in response in the two cerebral hemispheres. In the first part of the visual response (50–250 ms from stimulus onset), processing in each hemisphere was largely restricted to contralateral stimuli, with strong responses to such stimuli, and selectivity for both object and category. Later (300–500 ms), responses to ipsilateral stimuli also appeared, many cells now responding more strongly to ipsilateral than to contralateral stimuli, and many showing selectivity for category. Activity on error trials showed that late activity in both hemispheres reflected the animal's final decision. As information is processed towards a behavioural decision, its encoding spreads to encompass large, bilateral regions of prefrontal cortex.

## Introduction

Recordings from the lateral prefrontal cortex (LPFC) of behaving monkeys show extensive coding of visual information (e.g. Watanabe, 1986; Miller *et al*., 1996). An enduring question is the extent of regional specialisation for information of different kinds. An early, influential proposal held that ventrolateral prefrontal cortex (VLPFC) is specialised for coding of complex object information, while dorsolateral prefrontal cortex (DLPFC) processes spatial location (e.g. Goldman-Rakic, 1988; Wilson *et al*., 1993). Other kinds of clustering of the visual response within the LPFC have also been reported (see e.g. Ninokura *et al*., 2004). Certainly, different regions of LPFC have different patterns of anatomical connectivity. For example, complex visual information is transmitted to the VLPFC from inferotemporal cortex (Ungerleider *et al*., 1989), while DLPFC has greater connectivity to parietal and motor regions (Rushworth, 2000). Widespread connections within frontal cortex, however, suggest extensive transmission of information between regions (Pucak *et al*., 1996). Prominent models of cognitive control propose widespread broadcasting of information within and between the parts of an extended frontoparietal network (e.g. Goldman-Rakic, 1988; Dehaene *et al*., 2003). In line with widespread information exchange, many electrophysiological studies report substantial regional overlap for coding of different kinds of information (e.g. Watanabe, 1986; White & Wise, 1999; Wallis *et al*., 2001), and single cells with complex patterns of joint selectivity for several stimulus features (Rao *et al*., 1997; Tsujimoto *et al*., 2012; Rigotti *et al*., 2013).

In the present study, we used a cued target detection task to examine three kinds of visual information. Choice stimuli were pictures of objects, presented to left or right of fixation. Each trial began with an instruction cue indicating the target picture for this trial; if the subsequent choice stimulus was this target, the monkey was rewarded for a delayed saccade to its location, but otherwise held fixation. Using this task, we assessed prefrontal coding of object identity, behavioural category (target vs. nontarget) and location.

First, we compared activity in DLPFC and VLPFC, testing relative specialisation for location vs. object coding. Second, we compared responses to stimuli in the two visual fields, contralateral or ipsilateral to the recording area. While anatomical connections from visual to prefrontal cortex are largely intrahemispheric (Ungerleider *et al*., 1989), recording studies generally suggest only weak preference for stimuli in the contralateral hemifield (Boch & Goldberg, 1989; Rainer *et al*., 1998; Everling *et al*., 2002; Lennert & Martinez-Trujillo, 2013), probably reflecting information exchange between the two hemispheres (see e.g. Goldman-Rakic & Schwartz, 1982; Tomita *et al*., 1999). Third, given widespread connectivity within and between the two frontal lobes, we examined the time-course of information coding across regions and hemispheres. We addressed the hypothesis that, as stimulus processing develops, coding of task-critical information might become increasingly widespread.

In a recent report, data from this study were used to examine attentional competition between stimuli presented simultaneously to left and right (Kadohisa *et al*., 2013). Here we focus on coding of object, category and location for single, unilateral stimuli.

## Materials and methods

### Subjects

Subjects were two male rhesus monkeys (Macaca mulatta) weighing 11 (monkey A) and 10 (monkey B) kg. The experiments were performed in accordance with the Animals (Scientific Procedures) Act 1986 of the UK; all procedures were licensed by a Home Office Project License obtained after review by Oxford University's Animal Care and Ethical Review committee, and were in compliance with the guidelines of the European Community for the care and use of laboratory animals (EUVD, European Union directive 86/609/EEC).

### Task

**Fig. 1:**
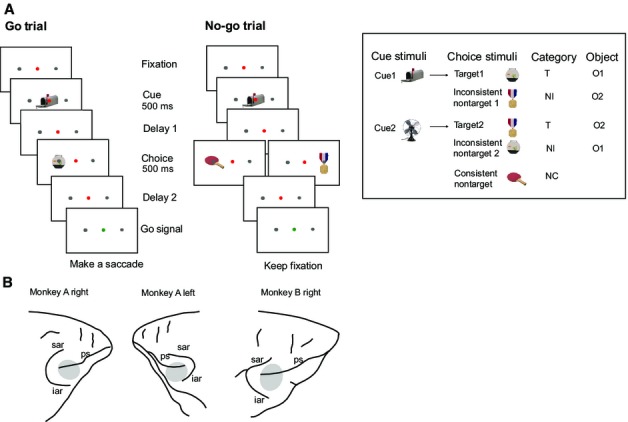
(A) Task. Following fixation on a central red dot, each trial began with a cue stimulus indicating the current target. For each animal, two alternative cues were associated with two alternative targets based on prior training (illustrated by stimuli for one animal in inset). Following an initial delay, the animal saw a choice display consisting of a single object to left or right of fixation. Possible display objects were the cued target (T), the object associated with the alternative cue (inconsistent nontarget, NI), and a third object never serving as a target (consistent nontarget, NC). Following a second delay, a change of fixation point to green instucted the animal to indicate his behavioural decision. At this point, for go trials (T present in choice display), the monkey was rewarded with a drop of liquid for a saccade to the T location. For no-go trials (T absent), reward was contingent on holding fixation until the end of the response interval (1000 ms; see Materials and Methods). Note that T following cue 1 is physically identical to NI following cue 2 (here labelled O1), while T following cue 2 is physically identical to NI following cue 1 (O2). (B) Recording locations in each recorded hemisphere. ps, principal sulcus; sar, superior arcuate sulcus; iar, inferior arcuate sulcus.

Trial events are illustrated in Fig. 1A. Each trial began with onset of a red dot at screen centre, which the animal was required to fixate (window 5° × 5° for monkey A, 4° × 4° for monkey B) until the final saccadic response at the end of the trial. A premature saccade outside the fixation window immediately terminated the trial without reward; aborted trials were discarded from all data analyses. After fixation had been held for 1000 ms, a central cue stimulus (500 ms, 2° × 2°) indicated the target for the current trial. Based on training before recordings began, each of two alternative cue stimuli was associated with a different target (see Fig. 1A inset for cue–target pairs for monkey A; different cue, target and nontarget images were used for monkey B). After cue offset there was a randomly varying delay of 400–600 (monkey A) or 400–800 (monkey B) ms, followed by a 500-ms choice display.

In the choice display, a stimulus object (2° × 2°) was displayed on the horizontal meridian randomly 6° to left or right of fixation. The stimulus object was either the cued target (T), the object associated with the alternative cue (inconsistent nontarget, NI), or a third object never used as a target (consistent nontarget, NC). Here we call these ‘behavioural categories’. Thus T following cue 1 was physically the same as NI following cue 2, while T following cue 2 was the same as NI following cue 1. Here the former is called object 1 (O1) and latter object 2 (O2). Note that, in addition to the single-object displays, there were also an equal number of two-object displays with simultaneous stimuli (one target and one nontarget or two nontargets) to left and right. Data from these two-object displays were the focus of our previous report (Kadohisa *et al*., 2013) and are not considered further here.

To avoid response biases, we adjusted frequencies of individual trial types to ensure that T was present in half of all single-object (and two-object) displays; otherwise, trial types appeared equally often and in random order. Following choice stimulus offset, there was a further delay of 100–150 (monkey A) or 300–500 (monkey B) ms, after which the fixation point turned green to indicate the monkey's response interval. For go trials (T present in choice display), the monkey was immediately rewarded with a drop of liquid for a saccade to the remembered T location (target window 6° × 6° for monkey A, 3.5° × 3.5° for monkey B). For no-go trials (T absent), the monkey was required to hold fixation for the whole 1000 ms response interval and was then either given immediate reward (monkey B) or was rewarded for a further saccadic response (monkey A). For monkey A, some sessions had cues randomly varying between trials, while others had alternating brief (15–20 trials) blocks of fixed cues. Physiological data were similar in the two cases and were combined. For monkey B, cues always varied randomly between trials.

### Recordings

Each monkey was implanted with a custom-designed titanium head holder and recording chamber(s) (Max Planck Institute for Biological Cybernetics), fixed on the skull with stainless steel screws. Chambers were placed over the lateral prefrontal cortex of the left [anterior–posterior (AP), 25.3; mediolateral (ML), 20.0] and right (AP, 31.5; ML, 22.5) hemispheres for monkey A and the right hemisphere (AP, 30.0, ML, 24.0) for monkey B. Recording locations are shown in Fig. 1B. A craniotomy was made under each chamber for physiological recording. All surgical procedures were aseptic and carried out under general anaesthesia. Data were recorded over a total of 140 daily sessions. We used arrays of tungsten microelectrodes (FHC, Bowdoin, ME, USA) mounted on a grid (Crist Instrument Co., Hagerstown, MD, USA) with 1 mm spacing between adjacent locations inside the recording chamber.

The electrodes were independently controlled by a digitally controlled microdrive (Multidrive 8 Channel System, NAN Instrument, Nazareth, Israel for monkey A; Multidrive 8 Channel System, FHC for monkey B). Neural activity was amplified, filtered, and stored for offline cluster separation and analysis with the Plexon MAP system (Plexon, Dallas, TX, USA). Eye position was sampled using an infrared eye tracking system (120 Hz, ASL, Bedford, MA, USA for monkey A; 60 Hz, Iscan, Woburn, MA, USA for monkey B) and stored for offline analysis. We did not preselect neurons for task-related responses; instead, we advanced microelectrodes until we could isolate neuronal activity before starting the task. At the end of the experiments, animals were deeply anaesthetized with barbiturate and then perfused through the heart with heparinized saline followed by 10% formaldehyde in saline. The brains were removed for histology and recording locations confirmed on dorsal and ventral frontal convexities and within the principal sulcus.

### Data analysis

Except for the specific analysis of error trials, physiological data were analysed just from successfully completed trials, on average including 17 repetitions for each combination of cue, choice stimulus type, and hemifield. All statistical analyses were done using MATLAB (MathWorks, Natick, MA, USA). For all analyses, spike data were smoothed with a Gaussian kernel of SD 20 ms, cutoffs ±1.5 SD.

## Results

As described in detail previously (Kadohisa *et al*., 2013), animals showed highly accurate responses to singly presented T stimuli (mean correct responses, 86% monkey A, 83% monkey B) and NC stimuli (83% monkey A, 87% monkey B), with more errors in response to NI (56% monkey A, 63% monkey B). Across the three recorded hemispheres (Fig. 1B), we isolated the activity of 145 single neurons from DLPFC (dorsal bank of principal sulcus and dorsolateral convexity; 62 neurons from monkey A right, 14 neurons from monkey A left, 69 neurons from monkey B right), and 274 neurons from VLPFC (ventral bank of principal sulcus and ventrolateral convexity; 115 neurons from monkey A right, 77 neurons from monkey A left, 82 neurons from monkey B right). Together these regions encompassed parts of areas 9, 46, 45A and 8A (Paxinos *et al*., 1999). We analysed responses to the singly presented choice stimuli illustrated in Fig. 1A, examining selectivity for behavioural category, object identity and stimulus location.

### Overview

**Fig. 2:**
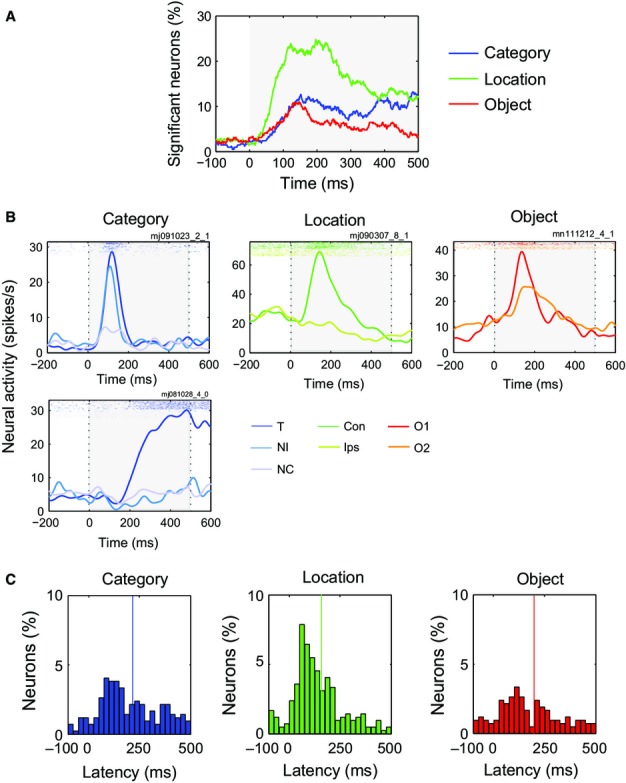
(A) Time-course of visual discriminations across the neural population. Percentage of all analysed neurons (*n* = 419) with significant discrimination of behavioural category (blue), object (red) and location (green), as a function of time from choice stimulus onset. Shaded area shows stimulus presentation interval. (B) Responses of example cells with category (left), location (middle) and object (right) selectivity. Rasters at top show spike trains on each individual trial. Con, contralateral hemifield; Ips, ipsilateral hemifield. (C) Latency distributions for category, location and object discrimination. Vertical lines show mean of all positive latencies.

To address the temporal evolution of category and location coding in the cell population, we separately examined the activity of each single cell using a series of anovas with factors behavioural category (T, NI, NC), stimulus location (contralateral or ipsilateral to recording site) and cue. Based on data smoothed as described above, anovas were performed for each separate 1-ms bin from −100 to +500 ms from choice stimulus onset. To minimise false positives, confirmed by low rates of significance prior to stimulus onset (Fig. 2A), coding of category and location were defined as significant throughout any time period of ≥ 30 ms during which the main effect passed a threshold of *P* < 0.05. Combining across dorsal and ventral regions, Fig. 2A shows percentages of all neurons showing significant main effects of category (blue) and location (green) in each time bin. Category coding in PFC began prior to 100 ms from stimulus onset, then climbed steadily to a level >10% of neurons by stimulus offset. Location coding was initially more prominent, reaching a peak above 20% of neurons at ∼ 200 ms, then somewhat declined. Fewer cells showed an interaction of behavioural category with location (mean 5.7% across the period 100–500 ms from stimulus onset).

Responses of two example category-selective cells are shown in Fig. 2B (left), one with an early, phasic response to T and NI, the other with late activity building up only for T. Responses of an example location-selective cell are shown in Fig. 2B (middle), with a strong response just to contralateral stimuli.

For each animal, two objects, O1 and O2, served as T and NI, changing behavioural category across trials. To examine coding of object identity rather than behavioural category we used anovas as before but with factors object (O1, O2), location and cue. Data from NC trials were omitted from this analysis. In Fig. 2A, the time-course of object coding (significant main effect of object) is shown in red. Unlike category coding, which increased throughout the stimulus period, object coding peaked at ∼150 ms, then declined. The data suggest early PFC coding of object identity, required to compute behavioural category, then progressive replacement by coding of the category itself. Again, fewer cells showed an interaction of object with location (mean 2.6% across the period 100–500 ms from stimulus onset). Responses of an example object-selective cell are shown in Fig. 2B (right).

Because of the smoothing kernel applied to spike data prior to analysis, discrimination latencies could only be approximately estimated. In particular, as data at each 1-ms timestep were influenced by spikes up to 30 ms later, latencies were likely to be underestimates. With this caveat in mind, for each cell with significant coding of category, location or object as determined in the above analyses, we defined the latency of discrimination as the start of the first significant period of coding. Histograms of these single-cell latencies are shown in Fig. 2C. In line with the results shown in Fig. 2A, latencies were shortest for location (mean ± SD, 154 ± 109 ms, negative latencies discarded), followed by object (198 ± 131 ms) and category (220 ± 131 ms).

Finally we asked whether category, location and object were coded by the same or different cells. For each cell, new anovas were used to measure category, location and object selectivity based on mean activity in two analysis windows, early (50–250 ms from choice stimulus onset) and late (300–500 ms). As measures of category, location and object selectivity in each cell, we used partial η^2^ for the corresponding main effect, calculated according to the standard formula:


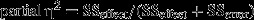


SS = sum of squares. Against proposals of specialisation, the three selectivity measures showed weak positive correlations across the cell sample, especially in the early window (correlations of 0.36, 0.33 and 0.20 for category–location, category–object and location–object, all *P* < 0.001), and to a degree also in the late window (correlations 0.25, 0.10 and 0.05, *P* < 0.001, *P* < 0.05, and *P* > 0.1 respectively).

In the following sections, we examine coding of category, object and location in more detail, comparing temporal evolution of coding for stimuli in contralateral and ipsilateral hemifields, and across dorsal and ventral regions.

### Coding of behavioural category

**Fig. 3:**
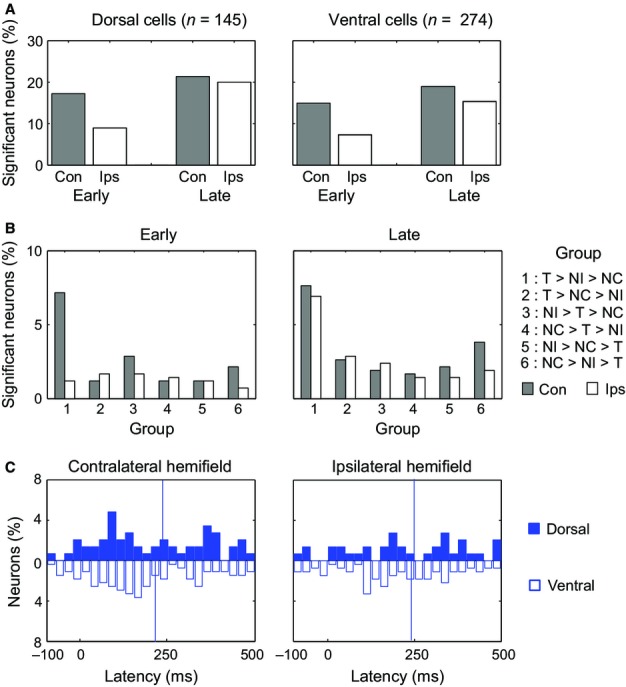
(A) Percentage of analysed neurons in each region (dorsal/ventral) with significant discrimination of behavioural category in each analysis window (early/late) for each hemifield. Early, 50–250 ms from stimulus onset; Late, 300–500 ms from onset; Con, contralateral hemifield; Ips, ipsilateral hemifield. (B) Percentage of all recorded neurons showing each possible pattern of stimulus preference. Data are shown separately for early and late analysis windows. (C) Latency distributions for category discrimination in each hemifield, separately for dorsal region (upper) and ventral region (lower). Vertical lines show mean of all positive latencies.

To examine category coding separately in contralateral and ipsilateral hemifields, data for each cell were examined with separate anovas for the two hemifields, each with factors category (T, NI, NC) and cue. To capture the temporal evolution of category coding, separate anovas for each cell were based on mean firing rates in early (50–250 ms) and late (300–500 ms) windows. Percentages of cells with a significant main effect of category (*P* < 0.05) are shown in Fig. 3A, with separate panels for dorsal and ventral cells.

Three findings are evident. First, in the early recording period there was strong side preference, with substantially more category coding of stimuli in the contralateral hemifield. In the early period, indeed, the percentage of cells with ipsilateral category coding was only slightly above the 5% expected by chance. Second, this side preference was abolished in the late period, with many cells now coding category on either side. Third, results were closely similar for dorsal and ventral cells, as regards both the level and pattern of category selectivity.

In a first set of tests, we used χ^2^ to compare proportions of significant category-coding cells in dorsal and ventral regions, with a separate test for each combination of hemifield and analysis window. In line with the pattern evident in Fig. 3A, no significant differences were found (maximum χ^2^_1_ = 1.5). We accordingly combined data from the two regions to test the frequency of contralateral vs. ipsilateral category coding, using McNemar's test. In the early period, contralateral coding was significantly more frequent (χ^2^_1_ = 14.5, *P* < 0.001) but in the late period this difference disappeared (χ^2^_1_ = 1.4).

Based on these same anovas for early and late periods, we proceeded to examine the fine structure of category coding. In previous data from a similar cued target detection task (Kusunoki *et al*., 2010), we found a preponderance of cells with a preference T > NI > NC, i.e. strongest response to the current target and weakest response to the stimulus always serving as a nontarget. In these previous data, the second most common pattern of preference was anti-target, i.e. NC > NI > T. For each cell showing significant category coding in one of the above four anovas (contralateral/ipsilateral × early/late), the pattern was classified into one of six groups: 1 (T > NI > NC), 2 (T > NC > NI), 3 (NI > T > NC), 4 (NC > T > NI), 5 (NI > NC > T), 6 (NC > NI > T). Percentages of cells in each group are shown in Fig. 3B. In line with our previous report, the most common pattern of preference was T > NI > NC. The exception was ipsilateral coding in the early window, where infrequent overall coding was accompanied by no evident pattern. To test the significance of these patterns, we took cells with highest response to T, and compared the frequencies of groups 1 and 2. Group 1 was more frequent for early contralateral data (χ^2^_1_ = 17.9, *P* < 0.001) and for both sides in the late period (χ^2^_1_ = 16.0 and 10.6 respectively for contralateral and ipsilateral, *P* < 0.001 and *P* < 0.002). The difference was not significant for early ipsilateral (χ^2^_1_ = 0.3). Similarly, among cells with weakest responses to T, we compared the frequencies of groups 5 and 6. These differences were not close to significant (maximum χ^2^_1_ = 1.6), showing little evidence of anti-target cells in the current data.

Finally, to examine the latency of category coding in each hemifield, we again analysed data at finer temporal resolution, with separate anovas on each 1-ms bin from −100 to +500 ms from stimulus onset, and latency defined as before. Distributions of latency are shown in Fig. 3C. The results confirm shorter latency for contralateral coding (dorsal cells, mean ± SD, 229 ± 142 ms; ventral cells, 215 ± 137 ms) than for ipsilateral coding (dorsal cells, 252 ± 144 ms; ventral cells, 240 ± 122 ms). Though there was a tendency for shorter latency in ventral cells, in line with our previous report (Kusunoki *et al*., 2010), this difference was far from significant (Mann–Whitney, contralateral *Z* = 0.44, ipsilateral *Z* = 0.49).

### Object coding

**Fig. 4:**
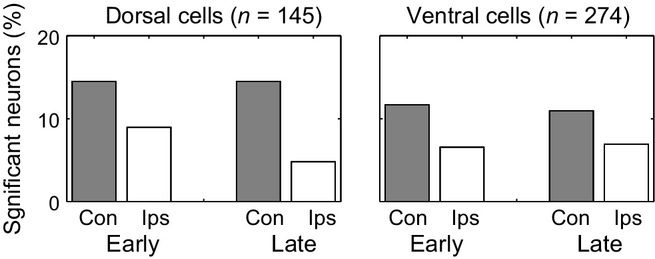
Percentage of analysed neurons in each region with significant discriminations of stimulus object in each analysis window for each hemifield. Conventions as Fig. 3A.

Separate anovas for early and late windows were also used to examine object coding in contralateral and ipsilateral hemifields, again separating data for dorsal and ventral cells. Again, these analyses concerned just those two objects for each animal that could serve as either T or NI, leaving out data for NC and sorting data according to factors of object (1, 2) and cue. Percentages of cells with a significant main effect of object (*P* < 0.05) are shown in Fig. 4, with separate panels for dorsal and ventral cells.

In this case the pattern was simple, with stronger object coding on the contralateral side in all cases. As before, initial tests showed no significant differences in the frequency of object coding between dorsal and ventral cells (maximum χ^2^_1_ = 1.1). Combining the two, contralateral coding was more frequent than ipsilateral in the early window (McNemar's test, χ^2^_1_ = 7.3, *P* < 0.01) and in the late window (χ^2^_1_ = 9.6, *P* < 0.01). Note that, with these broader time windows, there was less evidence for reduced object coding late in the stimulus period, as revealed in more time-resolved analysis (Fig. 2A).

### Location coding

**Fig. 5:**
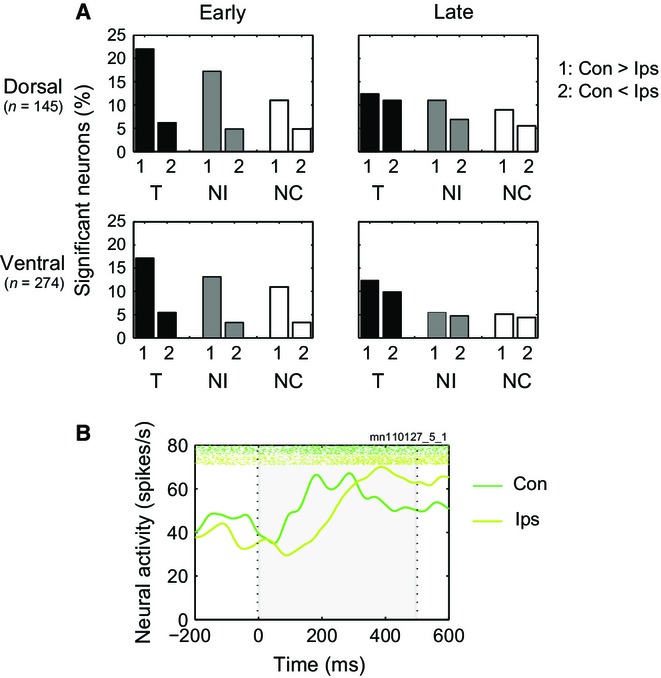
(A) Percentage of side-selective neurons for early and late analysis windows. For each behavioural category (T, NI, NC), histograms show percentage of neurons with significant difference Con > Ips (group 1) or Ips > Con (group 2); Con, contralateral hemifield; Ips, ipsilateral hemifield. (B) Activity of example neuron with varying side selectivity as a function of time from stimulus onset. Conventions as Fig. 2B.

Anovas on early and late windows were also used to examine the frequency of location coding in dorsal and ventral regions. As location was more relevant to the task for T stimuli, requiring a final saccade to the location where T had occurred, we carried out separate anovas for T, NI and NC trials. In each anova, factors were location (contralateral, ipsilateral) and cue. Results are shown in Fig. 5A. Again, results were very similar for dorsal and ventral regions. In the early window there was a strong predominance of contralateral preference but in the late window the frequency of contralateral preferences was reduced while the frequency of ipsilateral preferences increased, resulting in roughly equal numbers of cells with preferences for the two sides.

Again, initial tests showed no differences between dorsal and ventral cells (for each combination of behavioural category and analysis window, χ^2^ test on the relative frequency of contralateral preference, ipsilateral preference or no preference, maximum χ^2^_2_ = 5.4). Combining the two, χ^2^ tests showed significantly more contralateral- than ipsilateral-preferring cells in the early window (χ^2^_1_ = 29.4, 26.3 and 14.5 respectively for T, NI and NC; *P* < 0.001 in each case). In contrast, frequencies of contralateral and ipsilateral preference did not differ in the late window (maximum χ^2^_1_ = 1.2).

Figure 5B shows an example single cell that changed location preference from early to late periods. In the first phase of activity there was a strong response to contralateral stimuli, with no response or even weak suppression to ipsilateral (early period, contralateral > ipsilateral, *P* < 0.001 in anova with factors category × cue × hemifield). As this contralateral response declined, there was a sharp increase in response to ipsilateral stimuli, exceeding the contralateral response in the late period (ipsilateral > contralateral, *P* < 0.05).

### Error trials

To link neural activity to behavioural accuracy, we compared coding of behavioural category for correct and error trials. For this purpose we focused on two types of error: no-go responses to T stimuli, and go responses to NI stimuli. The high rate of the latter (see above) suggested failed use of cue information to define the current target. Separately for early and late analysis windows as previously defined, and for contralateral and ipsilateral stimuli, we selected all cells with a significant main effect of behavioural category (cf. Fig. 3A). Defining T_r_ and NI_r_ as the firing rates on T and NI trials respectively, for each cell we calculated the category index (T_r_ – NI_r_)/(T_r_ + NI_r_), separately for correct trials (T followed by go response, NI followed by no-go response) and error trials (T followed by no-go response, NI followed by go response).

**Fig. 6:**
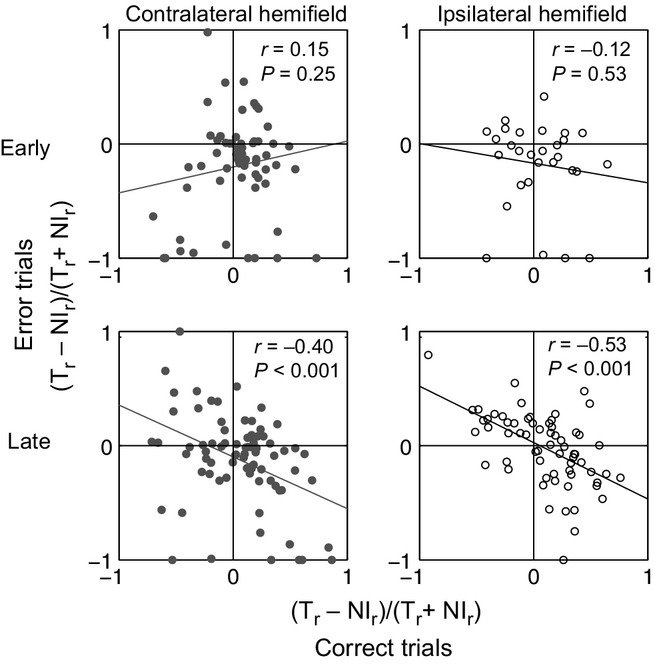
Scatterplots relating category index on correct and error trials. Each point shows data for a single cell.

Scatterplots relating the category index on correct and error trials are shown in Fig. 6. In early windows the two were rather unrelated, suggesting disruption of behavioural category coding on error trials. In late windows, in contrast, strong negative correlations emerged, as expected if late coding of behavioural category followed the monkey's decision rather than the stimulus presented. Results were very similar for ipsilateral and contralateral stimuli, linking late activity in both hemispheres to the animal's behaviour.

## Discussion

In line with previous reports, we found extensive coding of task-relevant visual information in the LPFC (e.g. Duncan, 2001; Miller & Cohen, 2001). Though cells were randomly selected across a large region of the LPFC, many showed significant coding of the location, object and category information required in the task. In this task, object identity was combined with the context provided by the prior cue to determine behavioural category and, correspondingly, we found that object coding showed an early peak and then declined, while category coding was sustained into the delay preceding the animal's final response (cf. Stokes *et al*., 2013). As required by the task, accordingly, early visual coding was replaced by coding of choice outcome. Across coding of object, category and location, response properties were strikingly similar in dorsal and ventral regions. There was no evidence for more frequent object coding in the VLPFC, or location coding in the DLPFC (cf. Rao *et al*., 1997; Rainer *et al*., 1998). Previously (Kusunoki *et al*., 2010) we reported a somewhat shorter mean latency for category coding in the VLPFC, but only a nonsignificant trend of this sort was found in the current data and, certainly, latency distributions for category coding strongly overlapped in the two regions. Though direct access to VLPFC and DLPFC may differ for object and location information, coding of these two stimulus features is widespread across the two regions from early in the visual response. These physiological results are in line with anatomical data, showing reciprocal connections between DLPFC and VLPFC (Yeterian *et al*., 2012).

Across the cell population, more broadly, we did not find any general tendency for different cells to code different stimulus features. Instead, there were weak positive correlations between one kind of selectivity and another. In line with many other reports, the dominant pattern is one of individual neurons combining variable degrees of selectivity for the different kinds of information required in the task (e.g. Rao *et al*., 1997; Rainer *et al*., 1998; Machens *et al*., 2005; Sigala *et al*., 2008; Tsujimoto *et al*., 2012; Rigotti *et al*., 2013).

In contrast to similar coding in dorsal and ventral cells, we found substantial differences in coding between contralateral and ipsilateral hemispheres. In the early period, up to ∼ 250 ms from stimulus onset, processing was focused in the hemisphere contralateral to the stimulus. In this period, responses were stronger, more object-selective and more category-selective for contralateral than for ipsilateral stimuli. Later this picture was substantially changed. With the arrival of ipsilateral information to each hemisphere, many cells now responded more strongly to ipsilateral than to contralateral stimuli, and category coding now emerged on both sides. The temporal specificity of contralateral dominance may partially explain variable and weak evidence for such dominance in previous studies (e.g. Boch & Goldberg, 1989; Rainer *et al*., 1998; Everling *et al*., 2002; Lennert & Martinez-Trujillo, 2013). Intriguingly, the appearance of late coding in the ipsilateral hemisphere was not seen for object information, suggesting that increasing information spread was predominantly concerned with the behaviourally critical final decision.

Most commonly, category-selective cells showed strongest response to targets (T), and weakest response to a stimulus (NC) that was never a target and thus easily classified as nontarget. These results resemble strong frontal responses to targets in prior single-unit (e.g. Kusunoki *et al*., 2010) and imaging (e.g. Jiang *et al*., 2000; Hon *et al*., 2006) studies. Though T cells were common, we have previously shown that they are not closely involved in the saccadic response itself (Everling *et al*., 2002; Kusunoki *et al*., 2009; Kadohisa *et al*., 2013). For example, they show little activity at the time of the saccade (Kadohisa *et al*., 2013) and are not active in control tasks requiring the same eye movement (Everling *et al*., 2002; Kusunoki *et al*., 2009), suggesting a role in task-specific behavioural categorization rather than direct saccade preparation. In a previous study, we found a second common group of cells with an anti-target pattern, firing most strongly to NC and least strongly to T, but in the present data, evidence for this anti-target pattern was weak or absent. Several differences between studies could explain this difference in results; in the previous study, for example, monkeys monitored a series of images awaiting the final target, meaning that any one image was most likely to be a nontarget.

The cells analysed here are a subset of those described in a previous report, examining attentional competition between simultaneous objects on the two sides (Kadohisa *et al*., 2013). In that report, we showed that early responses to a two-object display resemble those to the contralateral stimulus alone. Later, however, it is the most important stimulus (T) that dominates activity in both hemispheres (Kadohisa *et al*., 2013). Results for early activity complement the present demonstration that, in the early phase, stimulus properties are not well represented in the ipsilateral hemisphere, suggesting little access of visual information to this side. The Kadohisa *et al*. (2013) results for late activity suggest that, as information about two stimuli is exchanged between hemispheres, they compete for control of neural activity. The winner in this competition is determined by current behavioural significance, set in part by short-term context (T, NI) and in part by long-term learning (NC).

Dense connectivity between different regions of frontal cortex affords the opportunity for extensive information exchange, in line with theoretical models linking cognitive control to widespread information broadcasting in an extended frontoparietal network (Dehaene *et al*., 2003) The present results are in line with such broadcasting. Even early in stimulus processing, extensive coding of stimulus objects, behavioural categories and locations is found throughout the LPFC of the contralateral hemisphere. More slowly, coding of behavioural categories and locations develops also in the ipsilateral hemisphere, resulting in final widespread representation of important stimulus events.

## Abbreviations

DLPFC: dorsolateral prefrontal cortex
LPFC: lateral prefrontal cortex
NC: consistent nontarget
NI: inconsistent nontarget
O1: object 1
O2: object 2
T: target
VLPFC: ventrolateral prefrontal cortex
